# Predicting National Suicide Numbers with Social Media Data

**DOI:** 10.1371/journal.pone.0061809

**Published:** 2013-04-22

**Authors:** Hong-Hee Won, Woojae Myung, Gil-Young Song, Won-Hee Lee, Jong-Won Kim, Bernard J. Carroll, Doh Kwan Kim

**Affiliations:** 1 Samsung Biomedical Research Institute, Seoul, Korea; 2 Department of Psychiatry, Samsung Medical Center, Sungkyunkwan University School of Medicine, Seoul, Korea; 3 Mining Laboratory, Daumsoft, Seoul, Republic of Korea; 4 Laboratory Medicine and Genetics, Samsung Medical Center, Sungkyunkwan University School of Medicine, Seoul, Korea; 5 Pacific Behavioral Research Foundation, Carmel, California, United States of America; MIT, United States of America

## Abstract

Suicide is not only an individual phenomenon, but it is also influenced by social and environmental factors. With the high suicide rate and the abundance of social media data in South Korea, we have studied the potential of this new medium for predicting completed suicide at the population level. We tested two social media variables (suicide-related and dysphoria-related weblog entries) along with classical social, economic and meteorological variables as predictors of suicide over 3 years (2008 through 2010). Both social media variables were powerfully associated with suicide frequency. The suicide variable displayed high variability and was reactive to celebrity suicide events, while the dysphoria variable showed longer secular trends, with lower variability. We interpret these as reflections of social affect and social mood, respectively. In the final multivariate model, the two social media variables, especially the dysphoria variable, displaced two classical economic predictors – consumer price index and unemployment rate. The prediction model developed with the 2-year training data set (2008 through 2009) was validated in the data for 2010 and was robust in a sensitivity analysis controlling for celebrity suicide effects. These results indicate that social media data may be of value in national suicide forecasting and prevention.

## Introduction

Suicide is a leading cause of death worldwide. According to the World Health Organization, in the year 2020 approximately 1.53 million people will die from suicide. [1] As Durkheim established, suicide is not a mere individual phenomenon, but it is influenced by social and environmental factors. [2] These include economic indicators, social cohesion, publicized celebrity suicides, sunlight duration and temperature. [3]–[10] Although these studies found meaningful results, few of them examined the public mood state.

Recently, several studies investigated associations between suicide and consumer behaviors related to the public mood. [11]–[13] Studies on alcohol consumption and suicide suggest that population drinking tends to promote completed suicide. [13]–[15] A recent study in Taiwan found a correlation between suicide and lottery sales that was interpreted as reflecting hopelessness at the social level. [11] However, these consumer behaviors are at best indirect indicators of public mood.

Social media data such as weblog contents are more promising sources to gauge the public mood. [16]–[18] Despite the diversity of content at an individual level, the aggregate of millions of social media data points may provide a pragmatic representation of public mood. [18] Previous studies suggested new methods to measure national happiness by tracking the usage of key words among users of social media services. [19], [20] Moreover, it has been shown that online social media data can be used to predict changes in the stock market, [18] influenza infection rates, [21], [22] and box office receipts. [23] Therefore, social media data could be a promising source for investigating the association between suicide and public mood and for the refinement of suicide prediction models.

At 31 per 100,000, the annual suicide rate in South Korea is highest among the 30 Organization for Economic Cooperation and Development (OECD) countries as of 2009. [24], [25] In addition, South Korea is a global leader in internet infrastructure and usage. [26] These conditions enabled us to investigate suicide and social media data. Our primary hypothesis was that social media variables are meaningfully associated with nation-wide suicide numbers. Our secondary aim was to develop and test a national suicide prediction model that incorporates social media data. As described in Methods, we extracted two candidate variables from a very large body of social media postings. These two variables focused on the topics of suicide and dysphoria in the form of frequency among weblog entries.

## Materials and Methods

### Suicide Data

We obtained the number of completed suicide events in South Korea from January 1 2008 to December 31 2010. The data were thoroughly examined and verified by the Korea National Statistical Office (KNSO, http://kostat.go.kr/portal/english). Data for those years were considered because contemporaneous demographic and social media data were available. Data were extracted from death records defined as suicides according to the International Classification of Diseases-10 (ICD-10) codes X60–X84, which include suicides from all causes, including intentional self-poisoning and self-harm. [27] Monthly five year averages of suicide number from January 2003 to December 2007 also were computed so as to allow adjustment for seasonal variation. [28].

### Social Media Data

Daumsoft, one of the leading social media analysis and consulting firms in Korea, provided the social media data for the current study. The data were drawn from weblog posts in Naver blog (http://section.blog.naver.com), a weblog service offered by the biggest portal site in South Korea. A set of filtering operations was applied to exclude advertisements and other noisy texts for weblog posts written during the period between January 1, 2008 and December 31, 2010. The weblog service processed 153,107,350 posts on 5,093,832 registered weblogs during the above 3-year period.

To effectively simplify and quantify the enormous amount of social media data, we defined two measures: ‘suicide weblog count’ and ‘dysphoria weblog count’. The suicide weblog count was defined as the daily document frequency mentioning the Korean word *jasal* ‘suicide’ at least once. Similarly the ‘dysphoria weblog count’ was defined as the daily document frequency mentioning the Korean word *himdeulda*, which conveys the negative meanings ‘be tired’, ‘be painful’, or ‘be exhausted’ at least once. The word *himdeulda* was specifically chosen since it has co-occurred most frequently with *jasal* among the words expressing subjective psychological status of a writer or a speaker. The actual measures were obtained using SOCIALmetrics™, a social media analysis system offered by Daumsoft (http://www.daumsoft.com/eng/index.html). This system provides deep level keyword analysis and opinion mining for social media texts and other web documents.

### Economic and Meteorological Data

Economic and meteorological variables identified in previous studies of suicide were also considered. The economic data, [29], [30] including consumer price index, unemployment rate, and stock index valuations (Korea Composite Stock Price Index, KOSPI), were extracted from the KNSO. The meteorological data (sunlight hours and temperature) [9], [27] were obtained from the Korea Meteorological Administration (KMA, http://web.kma.go.kr/eng). Measurements from the observation station in Seoul were chosen as representative data.

### Data Reduction

**Figure 1:**
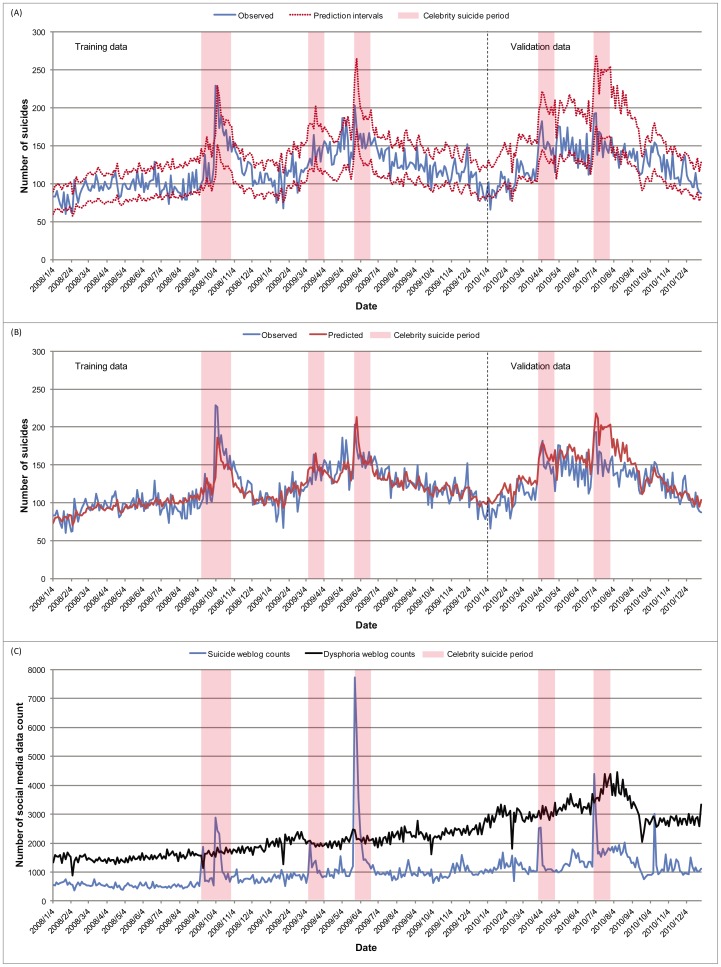
Prediction of nation-wide suicide number occurring in three-day epochs. Vertical bars denote the one month period following each celebrity suicide case (N = 6) (see Methods). These intervals overlapped for the first 2 celebrity suicide cases. Data of 2008 and 2009 were used as a training set and data of 2010 were used as a validation set. (**A**) Prediction range accuracy. Observed suicides (blue solid line) and prediction intervals (red dashed lines). The prediction range was computed for 85% probability. Prediction range accuracy was 0.88 for the training set and 0.79 for the validation set. (**B**) Predicted suicides (red) and observed suicides (blue). Correlations of 0.82 and 0.74 were obtained for 243 epochs of the training set and 121 epochs of the validation set, respectively. (**C**) Celebrity suicides and social media data. Suicide weblog counts (blue) and dysphoria weblog counts (black) are presented. The dysphoria weblog count was divided by 5 to adjust the ordinate axis scale with the suicide weblog count.

Prior to model construction, we divided the data into a 2-year training set (2008–2009) for identifying significant predictor variables and constructing a prediction model, and a 1-year validation set (2010) for evaluating the model. Most variables, including suicide counts and social media data, were summed in discrete 3-day epochs. There were 243 3-day epochs in the training set and 121 in the validation set. All computations were performed using these 3-day binned numbers. The end-of-week and holiday closing values of the KOSPI stock index were carried forward to the next active trading day. The most recent monthly data for the consumer price index and the unemployment rate were used each day, and these data were averaged for each 3-day epoch (see Text S1, Table A). For the meteorological variables we recorded averages of the 3-day data for temperature and sunlight hours in each epoch. For the purpose of illustration in Figure 1c, the dysphoria weblog count was divided by 5.

### Celebrity Suicides

In order to control for the influence of celebrity suicides, we noted the periods following these events. We defined celebrity suicide as a suicide exposed during more than two weeks in news programs of the three major national television networks (KBS, MBC and SBS). Six celebrity suicides met this definition during the 3 years of this study: actor (Jae-Hwan Ahn, 9/8/2008), actress (Jin-Sil Choi, 10/2/2008), actress (Ja-Yeon Jang, 3/7/2009), president (Moo-Hyun Roh, 5/23/2009), actor (Jin-Young Choi, 3/29/2010) and actor (Yong-Ha Park, 6/30/2010). Based on the study of Phillips, [31] the affected period was defined as a month (30 days) after the first report of the celebrity suicide. Prediction time points (3-day epochs) within or partly within this 30-day window were coded 1, while all others were coded 0 on the celebrity variable.

### Ethics Statement

Our research analyzes existing data and documents that are publicly available in a manner that does not allow individual subjects to be identified, therefore ethics approval was deemed unnecessary.

### Statistical Analysis

We identified significant variables by testing them individually in univariate linear regression analyses using the training set. The dependent variable (suicide numbers) was logarithm-transformed to satisfy a normal distribution assumption in the regression analysis. To avoid redundancy among the predictor variables, we chose the one variable with significance (P<0.05) and the highest adjusted R-squared value in each set of candidate variables that examined multiple time periods (t-1) to (t-5) (Text S1, Table A). In each case, this proved to be the most recent time period (t-1).

The multivariate regression model was constructed using these selected variables identified in the training set. Variables that became nonsignificant (P>0.05) were removed stepwise, so the final model included only significant variables. For all 3 years, we predicted suicide numbers by 3-day epoch using the ‘predict’ function with ‘prediction interval level’ set at 0.85 in the R software, which means that the observed number is expected to fall within the upper and lower boundaries of the prediction interval with 85% probability. Then, we compared the predicted numbers with the observed numbers. We regarded predictions as correct if the observed numbers fell within the prediction interval, and we defined the prediction accuracy as the ratio of correct predictions to total predictions. All statistical analyses including variable selection and model construction were performed using the R 2.9.1 public statistics software (http://www.r-project.org).

## Results

### Trend of National Suicide Numbers and Social Media Data

Over the 3 years of our study, national suicide numbers trended upwards (daily average: 105.7 in 2008, 126.9 in 2009, 127.7 in 2010), reflecting a general trend over 20 years preceding 2010 (Text S1, Figure A). Total suicides in 2010 were approximately 4.3 times the number in 1991. Daily suicide numbers varied substantially within each year, with a 5.4-fold range from a daily low of 16 to a daily high of 87 in 2008, a 4.6-fold range (18**–**83) in 2009 and a 3.5-fold range (20**–**69) in 2010. Spiking of completed suicides was apparent in proximity to celebrity suicides (Figure 1a, b).

Social media data also displayed marked variation. The dysphoria weblog count consistently exceeded the suicide weblog count. Daily suicide weblog counts ranged from a low of 104 to a high of 2,444 in 2008, 147 to 4,516 in 2009 and 186 to 3,588 in 2010; the daily dysphoria weblog counts ranged from 1,214 to 3,716 in 2008, 1,749 to 5,402 in 2009 and 2,400 to 7,668 in 2010. The suicide variable was more volatile or fluctuating than the dysphoria variable as evidenced by the coefficients of variation, defined as the ratio of the standard deviation to the mean. These were higher in daily suicide weblog counts than in dysphoria weblog counts (0.71 vs. 0.13 in 2008, 0.85 vs. 0.13 in 2009, and 0.53 vs. 0.16 in 2010). Spiking of suicide weblog counts was apparent in proximity to celebrity suicides, whereas no such short term spiking of the dysphoria weblog count was seen (Figure 1c).

### Univariate Analyses of Candidate Predictors of Suicide Numbers

Based on prior studies as reviewed above, we examined several candidate predictors of suicide numbers (Text S1, Table A). Univariate linear regression analyses confirmed the significance of all categories of variables except for sunlight duration. With the exception of the consumer price index, all the nominally significant variables displayed in Text S1 (Table A) remained highly significant (P<0.0002 or lower) after Bonferroni correction for multiple testing. Among the economic variables, the most significant were the unemployment rate and the stock market index. Rising unemployment and falling stock prices were each associated with higher suicide rates. The temperature data also showed a significant association with suicide data: increasing temperature was associated with higher suicide rates.

Among all the variables, ‘suicide (t-1)’, indicating the suicide number over the previous 3-day epoch, showed the most significant association, indicative of short term trending. This pattern is also seen extending back to 5 epochs (effectively 2 weeks). The significance of ‘suicide_5yr_avg’ suggests that there might be a monthly pattern concordant with a seasonal effect on suicides. [28] The ‘celebrity’ variable was confirmed as highly significant (Text S1, Table A and Figure 1a, b).

The social media variables also proved to be strongly associated with suicide numbers (Text S1, Table A)**.** Among the social media variables, the most recent suicide weblog count had the strongest association with completed suicide (P = 3.32×10^–15^). This variable displayed marked short term trends, with prominent spiking related to celebrity suicides (Figure 1c). By contrast, the dysphoria weblog count showed longer term secular trends and was not so obviously responsive to celebrity suicides in the short term (Figure 1c). Five variables showed a recency effect in the form of a declining strength of association extending back 2 weeks (t-5). These were suicide number, stock index, temperature, and the two social media variables (Text S1, Table A). The most significant variables from each set of candidate predictors are displayed in Table 1. These variables, that were the most significant in the univariate analyses, were used to develop the prediction model.

**Table 1:** Variables selected for multivariate prediction model development.

Variable	Description	*T*	*P*	AdjustedR-squared
**Suicide variables**				
suicide (t-1)	3-day sum of observed number of suicides at time t-1	16.40	<2.00×10^–16^	0.53
suicide_5yr_avg (t)	last five-year-average of suicides for the same month	4.74	3.60×10^–6^	0.08
**Economic, meteorological and celebrity variables**				
consumer price index (t-1)	change in monthly consumer price index from –13 monthsto –1 month	–2.89	0.004	0.03
unemployment (t-1)	monthly unemployment rate previous month	7.44	1.75×10^–12^	0.18
stock (t-1)	Korean stock index, KOSPI, most recent 3-day epoch average close	–7.11	1.29×10^–11^	0.17
temperature (t-1)	daily temperature, most recent 3-day epoch average	5.42	1.42×10^–7^	0.11
celebrity (t-1)	within one month after a celebrity suicidal event, 1; else, 0	8.45	2.80×10^–15^	0.22
**Social media variables**				
suicide weblog count (t-1)	most recent 3-day sum of weblog posts that contain the Koreanword *jasal* (meaning ‘suicide’) at least once	8.42	3.32×10^–15^	0.22
dysphoria weblog count (t-1)	most recent 3-day sum of weblog posts that contain the Koreanword *himdeulda* (meaning ‘be tired’, ‘be painful’, ‘difficult’or ‘be exhausted’) at least once	7.63	5.44×10^–13^	0.19

t indicates the predicted time point, and t-1 indicates a previous time point (see Methods for details). With the exception of the consumer price index, all variables were significant after Bonferroni correction for multiple testing (P<0.0002 or lower). The Table displays uncorrected *P* values.

### Prediction Model for Nation-wide Suicide Numbers

Using these selected variables, we constructed a prediction model for nation-wide suicide numbers (Table 2). Two classical economic variables, the consumer price index and the unemployment rate, were excluded in the final multivariate model as they became nonsignificant (P = 0.13 and 0.44, respectively). Within the final multivariate model, the ‘big three’ variables were the most recent suicide event rate, then the most recent dysphoria weblog count, then the most recent KOSPI stock market data. The celebrity variable and the most recent suicide weblog count were less powerful (Table 2). The final model showed an increased adjusted R-squared value (0.66) compared with the univariate models (0.03–0.53) and when compared with the multivariate model excluding social media data (0.59).

**Table 2:** Final variables included in the prediction model (adjusted R-squared = 0.66).

Variable	Regression coefficient	*t*	*P*
constant	4.07	34.83	<2.00×10^–16^
suicide (t-1)	0.003	6.27	1.74×10^–9^
dysphoria weblog count (t-1)	3.18×10^–5^	5.95	9.66×10^–9^
stock (t-1)	–2.30×10^–4^	–4.83	2.49×10^–6^
celebrity (t-1)	0.10	3.11	0.002
suicide_5yr_avg (t)	2.84×10^–4^	3.10	0.002
temperature (t-1)	0.004	3.00	0.003
suicide weblog count (t-1)	3.23×10^–5^	2.00	0.047

t indicates the predicted time point, and t-1 indicates a previous time point (see Methods for details).

### Validation of Model

When fitted to the training data set, the model showed a prediction accuracy of 0.88 (the observed numbers for 213 out of 243 3-day epochs ranged within the prediction interval), and in the validation set the prediction accuracy was 0.79 (96 out of 121 3-day epochs) (Figure 1a). The correlations of observed with predicted suicide numbers were 0.82 in the training set and 0.74 in the validation set (Figure 1b). Because of the obvious short term effect of celebrity suicides (Figure 1a–c), we conducted a sensitivity analysis of the model’s performance outside the celebrity suicide periods. No significant difference in performance of the model was seen in this secondary analysis: the prediction accuracy in the training set was 0.87 compared to the original accuracy of 0.88, and in the validation set the prediction accuracy was 0.83 compared with the original accuracy of 0.79; correlations of observed with predicted suicide numbers outside the celebrity suicide periods were 0.79 for the training data set and 0.72 for the validation data set. The corresponding original correlations were 0.82 and 0.74.

## Discussion

Social media variables were significantly associated with nation-wide suicide numbers. The suicide weblog count displayed the higher variability, especially in relation to celebrity suicide events. We interpret this finding as a reflection of short term reactivity and instability of prevalent affect at the social level, and it was associated with concurrent spiking of completed suicides. In contrast, the dysphoria weblog count showed longer term secular trends, and it was the more powerful of the two social media variables in the prediction model. We interpret this finding as a reflection of underlying mood at the social level. The distinction between transient affect and pervasive mood is well recognized at the individual level in clinical psychopathology. Our data suggest that this distinction is recapitulated at the social level.

We developed and validated a multivariate prediction model that combines the social media variables with other pertinent data. Our prediction model estimates suicide numbers in 3-day epochs with a reporting lag of one epoch. Moreover, the key variables in the prediction model appear to remain powerful over a period of 5 epochs (two weeks) before the index epoch (Text S1, Table A).

We knew in advance that the most important potential confound in our predictive model was likely to be the celebrity suicide contagion effect. Evidence of a significant impact of media reporting about celebrity suicides on short term suicide rates has been growing for decades. [32], [33] We confirmed this effect, and we found in addition that it now extends beyond the main stream media to social media. The suicide weblog count was responsive to celebrity suicide events in the short term, whereas the dysphoria weblog count did not obviously track celebrity suicide incidents (Figure 1c). Nevertheless, the dysphoria weblog count, which showed longer term secular trends, was by far the more powerful of the two social media prediction variables in the final model. The sensitivity analysis confirmed the robustness of the model outside celebrity suicide periods. Thus, our data indicate both short term and long term associations of social media variables with national suicide rate. Moreover, the social media data displaced some traditional economic predictors (consumer price index and unemployment rate) from the multivariate model. This finding suggests to us that the social media data reflect social mood and affect more directly than the economic data do. Our results suggest that it may now be feasible to consider the inclusion of social media data in surveillance of suicide trends. [34].

George and his colleagues suggested a concept of group affective tone that represents the collective affective reactions within a group. [35] Moreover, they showed the affective tone of a group was related to group behaviors such as cooperativeness. In this study, we found that the negatively valenced ‘dysphoria weblog count’ was significantly associated with nation-wide suicide number. This result suggests that the concept of group affective tone (i.e., mood) may be valid in a large population, with a significant and operationally detectable influence on the behavior of suicide.

Bollen and his colleagues developed a method to extract six mood dimensions from aggregated twitter contents. [36] In contrast, we used a large volume of weblog posts that contained specifically relevant keywords (‘suicide’ and its most related negative emotional word). Further study of the association between suicide and mood dimensions extracted from social media data would be valuable in order to explore the effects of diverse public mood states on suicide.

It has been proposed that the infectious disease model of contagion is useful for a conceptualization of suicide contagion. [36] In this context, we developed a prediction model of nation-wide suicide number with the methods employed in infectious disease prediction models. [21], [22] Thus, our data are consistent with a contagion effect of suicide exposure. [37] This effect was seen most obviously in the celebrity suicide periods.

We could not include some known variables that have an association with suicide, for example, day of the week, gaseous air pollutants level and allergen exposure. [38], [39] Previous studies of copycat suicides reported larger effects in youth and females. [40], [41] These results suggest that some population subgroups may be more affected by public mood. Further studies are required to investigate these issues.

In conclusion, we found a significant association of social media data with national suicide rate, resulting in a robust, proof-of-principle predictive model. Future models that build on this work and that incorporate social media data with other recognized social and economic predictors of suicide, may find application in forecasting and prevention of suicide. [42].

## Supporting Information

Figure A Trend of annual national suicide numbers per 100,000 persons in Korea, 1991–2010.
(PDF)

Text S1 **Table A.** Univariate regression analyses between individual variables and number of suicides at a prediction time point.
(DOC)
