# Supplementary figures and images for: Predicting National Suicide Numbers with Social Media Data
Source: PLoS One. 2013 Apr 22;8(4):e61809. doi: 10.1371/journal.pone.0061809 (PMC3632511; doi:10.1371/journal.pone.0061809)

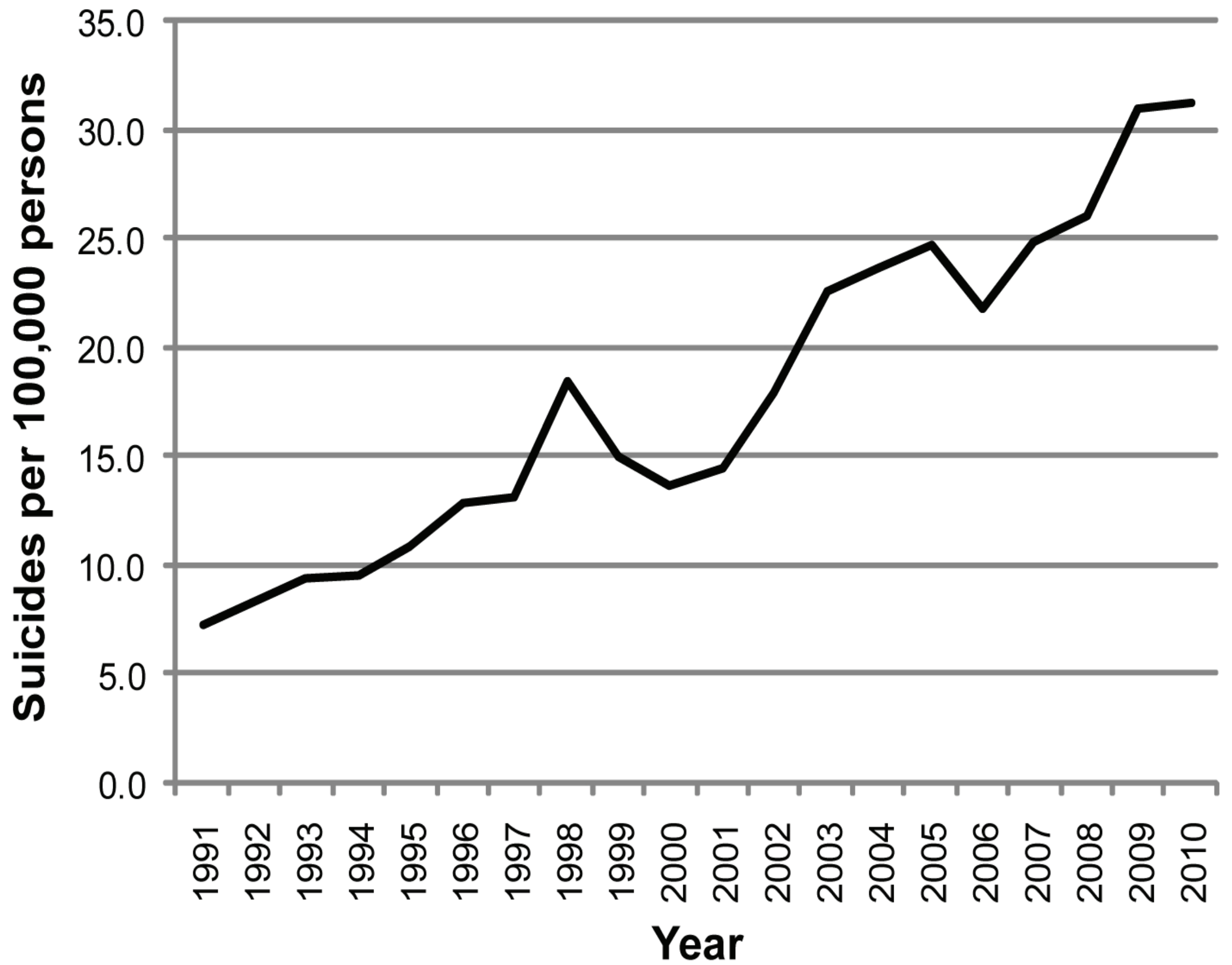

Figure A. Trend of annual national suicide numbers per 100,000 persons in Korea, 1991-2010.

Supplement: Figure A — Trend of annual national suicide numbers per 100,000 persons in Korea, 1991–2010. (PDF) [file pone.0061809.s001.pdf]
