# Supplementary material for: Predicting National Suicide Numbers with Social Media Data
Source: PLoS One. 2013 Apr 22;8(4):e61809. doi: 10.1371/journal.pone.0061809 (PMC3632511; doi:10.1371/journal.pone.0061809)
Supplement: Text S1 — Table A. Univariate regression analyses between individual variables and number of suicides at a prediction time point. (DOC) [file pone.0061809.s002.doc]

**Predicting national suicide numbers with social media data**

Hong-Hee Won^1^, Woojae Myung^2^**^*^**, Gil-Young Song^3^, Won-Hee Lee,^3^ Jong-Won Kim^4^,

Bernard J. Carroll^5^ & Doh Kwan Kim^2^**^*^**

^1^ Samsung Biomedical Research Institute, 50 Irwon-dong, Gangnam-gu, Seoul 135-710, Korea

^2^ Department of Psychiatry, Samsung Medical Center, Sungkyunkwan University School of Medicine, 50 Irwon-dong, Gangnam-gu, Seoul 135-710, Korea

^3^ Mining Laboratory, Daumsoft, 635-1 Hannam-dong, Yongsan-gu, Seoul, Republic of Korea

^4^ Laboratory Medicine and Genetics, Samsung Medical Center, Sungkyunkwan University School of Medicine, 50 Irwon-dong, Gangnam-gu, Seoul 135-710, Korea

^5^ Pacific Behavioral Research Foundation, Carmel, CA, USA

**^*^**These individuals contributed equally to this article as co-corresponding authors

**Supporting Information**

This supporting information has been provided by the authors to give readers additional information about their work.

**Table A. Univariate regression analyses between individual variables and number of suicides at a prediction time point.**

| **Variable** | **Description** | ***t*** | ***P*** | **Adjusted**  **R-squared** |
| --- | --- | --- | --- | --- |
| suicide (t-1) | 3-day sum of observed number of suicides at each time | 16.40 | <2×10^-16^ | 0.53 |
| suicide (t-2) |  | 16.00 | <2×10^-16^ | 0.51 |
| suicide (t-3) |  | 14.11 | <2×10^-16^ | 0.45 |
| suicide (t-4) |  | 13.16 | <2×10^-16^ | 0.42 |
| suicide (t-5) |  | 13.45 | <2×10^-16^ | 0.43 |
| suicide_5yr_avg (t) | last five-year-average of suicides for the same month | 4.74 | 3.60×10^-6^ | 0.081 |
| dysphoria weblog  count (t-1) | 3-day sum of weblog posts that contain the Korean word *himdeulda* (meaning ‘be tired’, ‘be painful’, or ‘be exhausted’) at least once | 7.63 | 5.44×10^-13^ | 0.19 |
| dysphoria weblog  count (t-2) |  | 5.272 | 3.00×10^-7^ | 0.10 |
| dysphoria weblog  count (t-3) |  | 6.704 | 1.44×10^-10^ | 0.15 |
| dysphoria weblog  count (t-4) |  | 5.597 | 5.96×10^-08^ | 0.11 |
| dysphoria weblog  count (t-5) |  | 6.318 | 1.29×10^-09^ | 0.14 |
| suicide weblog  count (t-1) | 3-day sum of weblog posts that contain the Korean word *jasal* (meaning ‘suicide’) at least once | 8.42 | 3.32×10^-15^ | 0.22 |
| suicide weblog  count (t-2) |  | 7.88 | 1.16×10^-13^ | 0.20 |
| suicide weblog  count (t-3) |  | 7.06 | 1.84×10^-11^ | 0.17 |
| suicide weblog  count (t-4) |  | 6.95 | 3.47×10^-11^ | 0.17 |
| suicide weblog  count (t-5) |  | 6.18 | 2.71×10^-9^ | 0.14 |
| consumer price index (t-1) | change in monthly consumer price index from – 13 months to – 1 month | -2.89 | 0.004 | 0.03 |
| unemployment (t-1) | monthly unemployment rate previous month | 7.44 | 1.75×10^-12^ | 0.18 |
| stock (t-1) | 3-day average of Korean stock index (KOSPI) closing at each time | -7.11 | 1.29×10^-11^ | 0.17 |
| stock (t-2) |  | -7.06 | 1.82×10^-11^ | 0.17 |
| stock (t-3) |  | -6.95 | 3.39×10^-11^ | 0.16 |
| stock (t-4) |  | -6.93 | 3.83×10^-11^ | 0.16 |
| stock (t-5) |  | -6.90 | 4.80×10^-11^ | 0.16 |
| sunlight (t-1) | 3-day average sunlight duration at each time | 0.71 | 0.482 | -0.002 |
| sunlight (t-2) |  | 1.37 | 0.171 | 0.004 |
| sunlight (t-3) |  | 0.65 | 0.516 | -0.002 |
| sunlight (t-4) |  | 0.74 | 0.459 | -0.002 |
| sunlight (t-5) |  | 1.26 | 0.208 | 0.002 |
| temperature (t-1) | 3-day average daily temperature at each time | 5.42 | 1.42×10^-7^ | 0.11 |
| temperature (t-2) |  | 4.57 | 7.90×10^-6^ | 0.08 |
| temperature (t-3) |  | 4.17 | 4.35×10^-5^ | 0.06 |
| temperature (t-4) |  | 4.56 | 8.39×10^-6^ | 0.08 |
| temperature (t-5) |  | 4.51 | 1.02×10^-5^ | 0.08 |
| celebrity (t-1) | within one month from a celebrity suicidal event, 1; else, 0 | 8.45 | 2.80×10^-15^ | 0.22 |

t indicates the predicted time point, and t-1, t-2, etc. indicate previous time points (see Methods for details).

*P* values are uncorrected for multiple comparisons (N = 34).
